# Achromobacter xylosoxidans/denitrificans Bacteremia in a Patient With Good’s Syndrome

**DOI:** 10.7759/cureus.13827

**Published:** 2021-03-11

**Authors:** Saiara Choudhury, Sudhakar Papineni, Srikanth Ramachandruni, Jurairat Molina, Salim Surani

**Affiliations:** 1 Internal Medicine, Corpus Christi Medical Center, Corpus Christi, USA; 2 Internal Medicine, University of North Texas, Dallas, USA

**Keywords:** good’s syndrome, hypogammaglobulinemia, thymoma, opportunistic infections, immunodeficiency

## Abstract

Good’s syndrome is a rare, adult-onset immunodeficiency syndrome associated with thymomas. Here, we present a 71-year-old, previously healthy male with a history of opportunistic infections status post-thymectomy, who presented with a septic knee complicated with bacteremia without any inciting factor. Therefore, a diagnosis of Good syndrome was made. While this is a rare disease, clinicians should have a high suspicion in patients with a history of thymoma. Early diagnosis and treatment can reduce opportunistic infections and improve morbidity and mortality.

## Introduction

Good’s syndrome is a rare, adult-onset, T-cell immunodeficiency syndrome associated with thymomas [1]. It was first described by Good in 1954 and characterized as adult-onset immunodeficiency with altered cell-mediated immunity, low to non-circulating B cells, and thymoma [2]. The thymus gland is a lymphatic organ that has many immunologic functions, including the development of immunocompetent T cells, the differentiation and proliferation of naive T-cells into cluster of differentiation 4 (CD4) T helper cells and CD8 cytotoxic suppressor cells, migration of mature T cells into the circulating lymphocyte pool and peripheral tissues. The thymus gland also plays a role in inducing immune self-tolerance and preventing autoimmunity [3]. Benign epithelial thymomas are the most common neoplasm arising from the thymus gland, making up almost 20%-25% of all mediastinal tumors and 50% of all anterior mediastinal masses [3]. Thymomas are commonly associated with autoimmune disorders like myasthenia gravis (30%-50%), systemic lupus erythematosus, pure red cell aplasia, syndrome of an inappropriate diuretic hormone, bullous pemphigoid, dermatomyositis, ulcerative colitis, and rheumatoid arthritis [3]. Good’s syndrome commonly manifests in patients between 40 and 70 years of age with presenting symptoms of recurrent opportunistic infections with viruses, fungus, and encapsulated bacteria [4]. While there have been multiple proposed hypotheses for the pathophysiology of the disease, it remains largely unknown. Treatment usually involves surgical resection of the thymic mass, antibiotic therapy for infection, intravenous immunoglobulin (IVIG), and antiviral prophylaxis, if needed [1-2,4]. Even though this syndrome is rare, it should be included in the differential diagnosis of immune deficiency since a delayed or missed diagnosis may lead to high morbidity and mortality [2].

## Case presentation

**Figure 1:**
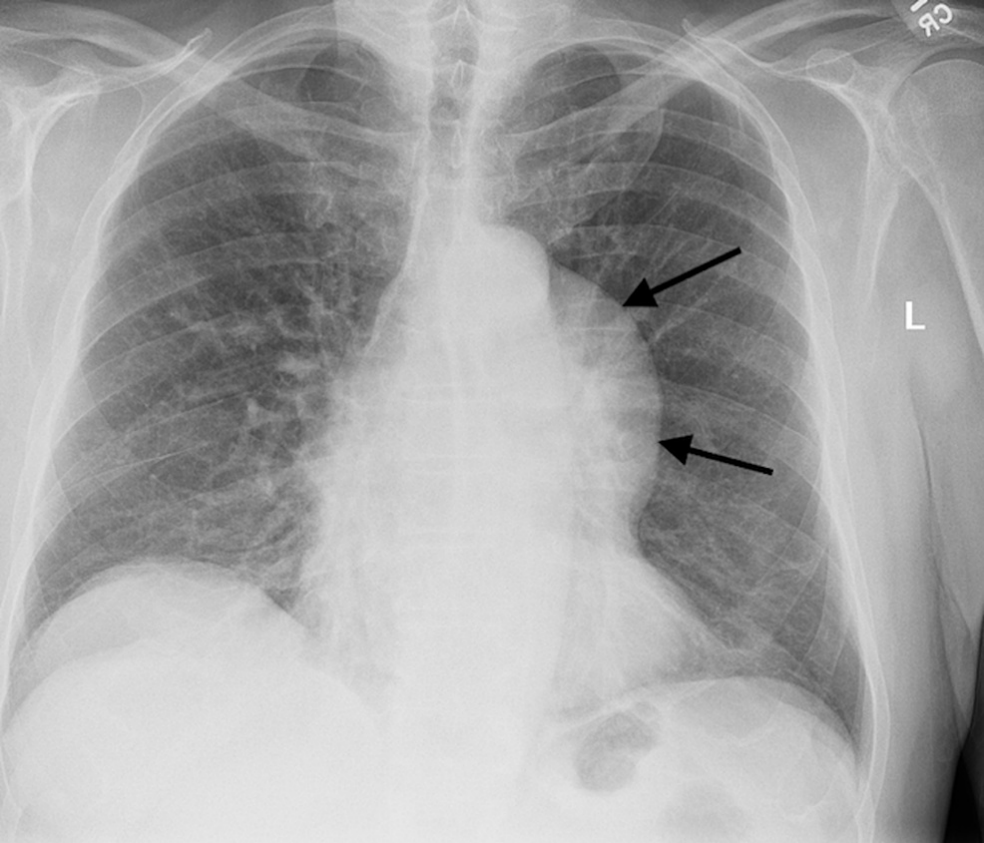
Posteroanterior (PA) chest radiograph of the anterior mediastinal mass (as indicated by the arrows). It is identified by the hilum overlay and preservation of posterior mediastinal lines.

**Figure 2:**
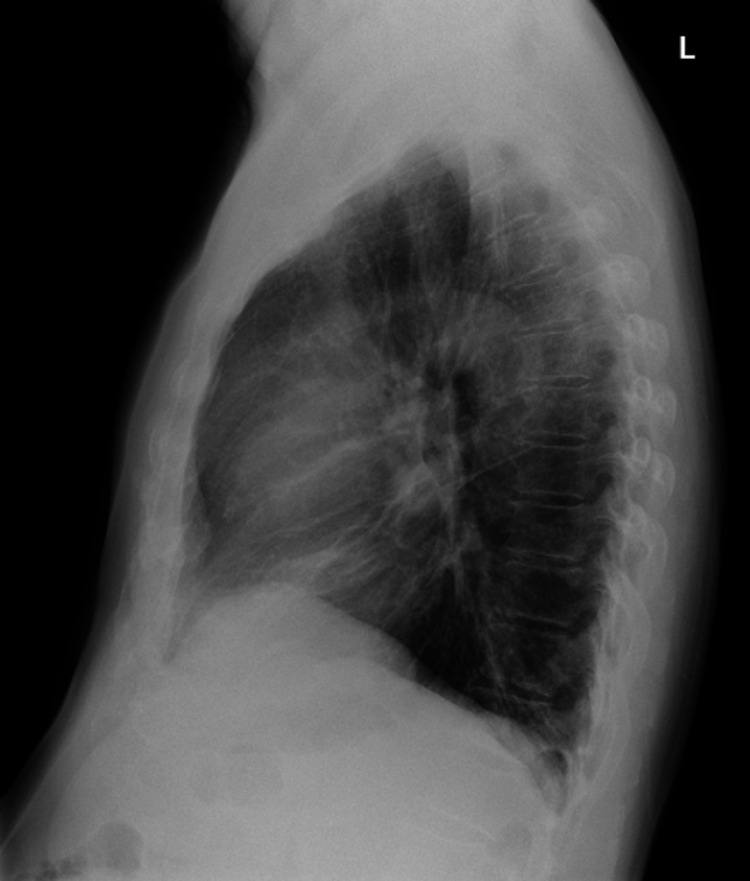
Lateral chest radiograph of anterior mediastinal mass

A 71-year-old male, with a past medical history of chronic obstructive pulmonary disease (COPD), anterior mediastinal mass found to be a type AB thymoma (pre-thymectomy images shown in Figures 1-2) status post-resection six months prior to this hospital admission, and a history of pneumonia and bacteremia with both the bronchoalveolar lavage (BAL) and blood cultures positive for *Pasteurella multocida *five months prior to this admission, presented with a three-week history of worsening pain, erythema, swelling and redness of right knee. It was associated with low-grade fevers, chills, and inability to bear weight on the right leg. He denied any recent trauma, falls, surgeries, or insect bites to the knee. The patient had come to the emergency department three days prior with a similar complaint of right knee pain. Blood cultures and arthrocentesis cultures from that visit were positive for *Achromobacter xylosoxidans/denitrificans*. The patient was notified by the hospital and asked to come back to the hospital for antibiotic therapy. On the day of admission, the radiograph of the right knee was negative for osseous abnormalities. The patient underwent debridement of the right knee joint by orthopedic surgery. Intraoperative wound cultures and repeat blood cultures were positive for *Achromobacter xylosoxidans/denitrificans*.

The patient reported he was healthy before the thymoma resection. The patient underwent a workup to investigate for underlying immunodeficiency and autoimmune disease. Human immunodeficiency virus (HIV) screen, rapid plasma reagin (RPR), acute hepatitis panel, rheumatoid factor, antinuclear antibody (ANA), and acetylcholine receptor antibody were negative. Peripheral blood smear showed a slightly diminished white count with relative monocytosis without blasts, normocytic normochromic anemia without schistocytes, and adequate platelet count. Immunoglobulin panel was done, which showed immunoglobulin G (IgG) <30 (reference range 603-1616 mg/dL), IgG1 <14 (reference range 248-810 mg/dL), IgG2 4 (reference range 130-555 mg/dL), IgG3 <1 (reference range 15-102 mg/dL), IgG 4 <1 (reference range 2-96 mg/dL), IgA <5 (reference range 61-437 mg/dL), and IgM <5 (reference range 15-143 mg/dL). Absolute CD4 count was 117/uL (359-1519), absolute CD8 count was 73/uL (109-897), and T lymphocyte CD4/CD8 ratio was 1.56. A two-dimensional (2D) echocardiogram showed normal systolic function with an ejection fraction of 60%-64%, without any obvious vegetation or thrombus. CT scan of the abdomen/pelvis with intravenous contrast was unremarkable.

Given the history of thymoma with severe immunodeficiency, a diagnosis of Good’s syndrome was made. Infectious disease was consulted, and the patient was started on antibiotic therapy with intravenous (IV) meropenem. He was given one dose of intravenous immunoglobulin (IVIG) in the hospital and developed an anaphylactic reaction requiring one night stay in the intensive care unit for supportive management and monitoring. The patient was discharged to a skilled nursing facility with six weeks of intravenous antibiotic therapy and physical therapy. He was arranged to follow up with an allergy and immunology specialist outpatient for further management of Good’s syndrome with IVIG.

## Discussion

While Good’s syndrome was first described some time ago, there have only been a few hundred cases reported in the literature. The study by Malphette et al. showed that usually patients with Good’s syndrome were diagnosed in their 50s-60s, without any significant gender predominance [5]. Diagnosis of thymoma was usually observed before a diagnosis of hypogammaglobulinemia, which was the case for our patient as well. About 35% of thymomas remain asymptomatic while 40% have local symptoms leading to further investigation. Our patient’s diagnosis was made when he was found to have recurrent dry cough and dysphagia for two months and had otherwise negative workup. Other symptoms of thymoma may include chest pain, shortness of breath from compression of the airways or the neuromuscular effects of myasthenia gravis, superior vena cava syndrome, and sometimes unexplained weight loss [6]. The presence of thymoma may be associated with disorders like myasthenia gravis, pure red cell aplasia, parathyroid adenoma, and hypogammaglobulinemia [6]. Our patient’s chest radiograph showed an anterior mediastinal mass, which was resected by thoracic surgery. Pathology showed benign type AB thymoma with clear margins. Therefore, no further treatment with chemotherapy or radiation was needed.

Two months after his thymectomy, the patient was admitted to the hospital with septic shock with bacteremia and severe bilateral pneumonia due to *Pasteurella multocida*. *Pasteurella multocida* is a rare pathogen that causes bacteremia, so an extensive literature review was conducted. Limited cases of bacteremia due to *Pasteurella multocida* were observed in patients who were usually immunocompromised due to malignancies, systemic infection, end-stage renal disease, cardiac disease, hypertension, diabetes, COPD, cirrhosis, or advanced age [7-8]. While our patient had COPD and had a cat in the house, which was thought to be the source of his bacteremia, this was unusual for someone who had been otherwise healthy. Although the patient was appropriately treated with antibiotics, no immunological workup was done at that time resulting in a delay in the diagnosis of Good’s syndrome.

During this admission, the patient presented with a septic knee and bacteremia due to *Achromobacter xylosoxidans/denitrificans* without any inciting traumatic event.​​​​​ It is an aerobic motile oxidase-positive, gram-negative bacillus that was first described in 1971 from chronic, purulent otitis media [9]. This organism is a very rare cause of bacteremia. There has only been a limited number of cases reported of *Achromobacter​​​​​​​* *xylosoxidans/denitrificans* causing bacteremia and the majority of the patients were severely immunocompromised with malignancies, renal or cardiovascular disease, history of intravenous drug use, and history of the prosthetic valve with endocarditis [9-10]. Due to the history of recurrent infections with uncommon organisms, immunologic and autoimmune workup was done, which showed hypogammaglobulinemia with CD4 lymphopenia. These are characteristic findings in addition to thymoma in patients with Good’s syndrome and thus the diagnosis was made [5].

In a study by Malphette et al., 95% of the patients who were found to have Good’s syndrome developed infectious complications, including pneumonia, recurrent bronchitis, and sinusitis, chronic diarrhea secondary to *Campylobacter*, *Shigella*, or *Salmonella* and 38% of the patients developed opportunistic infections such as oral thrush, esophageal candidiasis, cytomegalovirus (CMV)-associated disease, aspergillosis, and even disseminated *Mycobacterium tuberculosis* [5]. Cases have also been reported of patients with Good’s syndrome with babesiosis, Kaposi's sarcoma, CMV retinitis, Herpes vegetans, and Erythema multiforme [4,11-13], however, our patient may be the first reported case of Good’s syndrome with bacteremia due to *Achromobacter*​​​​​​​* xylosoxidans/denitrificans. *

While the pathophysiology of immunodeficiency remains elusive, there have been some proposed hypotheses. In a recent study by Guevara et al., it was hypothesized that the underlying mechanism of Good’s syndrome may be due to the improper differentiation of plasma cells, loss of naive memory of the CD4+ T cell population that may lead to defects in cell-mediated immunity and increase susceptibility to infections and anti-cytokine antibodies (mainly interferon (IFN) α, IFN-β, interleukin (IL) 1α, IL-12, IL-17A) that may have a negative effect on the growth and differentiation of pro B lymphocytes [14].

Additionally, it is also important to note that, in the study by Malphette et al., 75% of patients with Good’s syndrome had autoimmune conditions, including oral lichen planus, autoimmune hemolytic anemia, inflammatory bowel disease, and neutropenia [5]. Many of these autoimmune conditions persisted or were diagnosed after thymectomy. The study also showed that unlike common variable immunodeficiency disease, Good’s syndrome was not associated with lymphoid hyperplasia, leukemia, or lymphoma [5]. Hence, it is important to keep in mind for patients with thymoma who are being worked up for Good’s syndrome, as they may benefit from autoimmune workup as well.

Treatment of this disease usually involves resection of the thymoma, proper targeted antibiotic therapy in patients with underlying infection, and a thorough immunological workup. In patients with global hypogammaglobulinemia, IVIG is indicated [4-5,14]. Our patient posed a further complication, as he developed a severe adverse reaction to the first dose of IVIG. Adverse reaction to IVIG in our patient seems to be immediate, as it occurred during the infusion. We thought that this reaction is rate-related since true IgE-mediated anaphylaxis usually occurs hours after the infusion. However, a true IgE mediated anaphylaxis is possible especially in an IgA-deficient patient, which is in our case. A careful selection of IG preparations and pre-medications with antihistamines, acetaminophen, and corticosteroid were critical in further treatment of this patient [15]. Thus, patients with Good’s syndrome must have appropriate follow-up arranged with an immunologist after the acute infection is appropriately treated for necessary immunological interventions, including immunoglobulin infusion and a proper immunization plan that excludes the use of live vaccines.

## Conclusions

Good’s syndrome is a rare condition, and a high degree of suspicion is needed for the early diagnosis of this disease. In patients with a benign thymoma, it is critical to recognize the possibility of concomitant immunodeficiency and autoimmunity, and these should be thoroughly worked up in an interdisciplinary manner. These patients are also very susceptible to bacterial, viral, fungal, and opportunistic infections and should be appropriately immunized.
